# Overexpression of GOLPH3 is associated with poor prognosis and clinical progression in pancreatic ductal adenocarcinoma

**DOI:** 10.1186/1471-2407-14-571

**Published:** 2014-08-07

**Authors:** Luan-Jing Zhang, Ke-Bing Wang, Long-shan Liu, Lian-zhou Chen, Bao-Gang Peng, Li-Jian Liang, Zhi Li, Ling Xue, Wen Li, Jing-Tang Xia

**Affiliations:** Laboratory of General Surgery, The First Affiliated Hospital, Sun Yat-sen University, Guangzhou, Guangdong 510080 China; Department of General Surgery, The third Affiliated Hospital, Guangzhou Medical University, Guangzhou, Guangdong 510150 China; Department of Hepatobiliary Surgery, The First Affiliated Hospital, Sun Yat-sen University, Guangzhou, Guangdong 510080 China; Department of Pathology, The First Affiliated Hospital, Sun Yat-sen University, Guangzhou, Guangdong 510080 China

## Abstract

**Background:**

Golgi phosphoprotein 3 (GOLPH3) has been identified as an oncoprotein in various human cancers; however, its role in pancreatic ductal adenocarcinoma (PDAC) is unknown. We examined GOLPH3 expression levels and relationship with survival in patients with PDAC to establish the significance of GOLPH3 in the development and progression of PDAC.

**Methods:**

Real-time qPCR and Western blotting were performed to analyze the expression levels of GOLPH3 mRNA and protein in paired PDAC tumor and adjacent non-tumor tissues. Immunohistochemistry was used to analyze the expression levels of GOLPH3 protein in paraffin-embedded tissues from 109 cases of PDAC. Univariate and multivariate analyses were performed to identify correlations between the immunohistochemical data for GOLPH3 expression and the clinicopathologic characteristics in PDAC.

**Results:**

Expression levels of GOLPH3 mRNA and protein were upregulated in PDAC lesions compared to paired adjacent noncancerous tissues. Expression of GOLPH3 was significantly correlated with clinical stage (*P* = 0.006), T classification (*P* = 0.021), N classification (*P* = 0.049) and liver metastasis (*P* = 0.035). Patients with high GOLPH3 expression had shorter overall survival times compared to those with low GOLPH3 expression (*P* = 0.007). Multivariate analysis revealed that GOLPH3 overexpression was an independent prognostic factor in PDAC.

**Conclusions:**

Our findings suggest that GOLPH3 expression status may be a potential prognostic biomarker and therapeutic target in PCAC.

## Background

Pancreatic ductal adenocarcinoma (PDAC) is the fourth leading cause of cancer-related death in Western countries and the sixth in China [1, 2]. The mortality rates of PDAC closely equal its incidence [3], and the overall 5-year survival rate in patients with PDAC after diagnosis is less than 5%, with no apparent improvement over the past 25 years [4, 5]. Although surgical resection is currently the only potentially curative option in patients with PDAC, only 15%–20% of patients have resectable disease, and only around 20% of those survive to 5 years [6, 7]. However, detailed staging, patient selection, a standardized operative approach and routine use of multimodality therapies have contributed to an increase in the 5-year survival rate (actual 27%) in patients with resected PDAC [8].

Translational research into the molecular biology of pancreatic cancer has led to important advances in early diagnosis, the assessment of prognosis, and better disease management [3, 9]. In this study we investigated and identified Golgi phosphoprotein 3 (GOLPH3) as potential prognostic and predictive marker associated with poor survival rates in PDAC. Our aim is to find novel effective therapeutic targets and improve treatment outcome in patients with PDAC.

Golgi phosphoprotein 3 (GOLPH3), also known as GPP34, GMx33, MIDAS and yeast Vps74p, is a cytosolic trans-Golgi-associated protein with molecular weight of 34 kDa. GOLPH3 was initially identified through proteomic analysis of rat liver Golgi protein, and has been found to play important roles in protein sorting, receptor recycling and glycosylation [10–13]. More recently, GOLPH3 has been identified as a novel oncogene in various cancer types [14]. Overexpression of GOLPH3 has been reported in breast cancer [15], esophageal squamous cell cancer [16], oral tongue cancer [17] and glioblastoma multiforme [18]. Golph3 gene is located on chromosome 5p13 and is highly conserved in eukaryotic cells from yeast to humans [12]. Amplification of GOLPH3 at 5p13 has been reported in diverse solid tumors, including lung, ovarian, breast, prostate, melanoma and pancreatic cancer. GOLPH3 enhances growth-factor induced mTOR signaling and modulate the response to rapamycin [19]. Those investigations have uncovered some potential links of GOLPH3 with cellular function to tumorigenesis which is very important for us to further understand how this protein contritute to cancer pathology. Untill now the significance of GOLPH3 in PDAC is unknown. Therefore, we examined GOLPH3 expression in 109 cases of formalin-fixed, paraffin-embedded (FFPE) tissue specimens of human PDAC, and performed univariate and multivariate analyses to correlate its expression levels with patient survival and clinicopathologic features in PDAC.

## Methods

### Patient treatments and PDAC tissue specimens

Archived and formalin-fixed paraffin-embedded (FFPE) tissue samples were obtained from 109 patients diagnosed with PDAC, who had undergone surgical resection or biopsy between September 2003 and March 2011 in the Department of Hepatobiliary Surgery, the First Affiliated Hospital of Sun Yat-sen University, China. Initial radical resection had been performed on 69 patients and 40 patients received palliative surgery. All of the patients received ultrasound and computed tomography scans prior to surgery. Chemotherapy was administered postoperatively to 24 patients with advanced stage of PDAC. None of the patients received radiotherapy. Median follow-up time of surviving patients was 8.3 months (range, two days to 63.5 months).

Four matched pairs of fresh PDAC tumor and adjacent no cancerous tissue samples(at less 2 cm away from the margin of tumor tissue) were also obtained for testing the mRNA and protein levels of GOLPH3 expression. Histopathology analysis with HE staining on frozen sections had confirmed that the tumor tissues comprised of >70% cancer cells without necrosis, and that no cancer lesions were present in the no cancerous tissues.

The study was approved by the Medical Ethical Committee of the First Affiliated Hospital, Sun Yat-sen University (Guangzhou, China). Informed consent had been obtained from all of the patients for use of the clinical specimens.

### RNA extraction and real-time qPCR

Total RNA from the primary tumor and adjacent non-tumor tissue samples were extracted using Trizol reagent (Invitrogen; Carlsbad, CA, USA) according to the manufacturer’s instructions. The RNA was pretreated with RNAase-free DNase, and 2 μg RNA from each sample was used for cDNA synthesis. Real-time qPCR was performed using a Bio-Rad CFX96 Sequence Detection system (Bio-Rad; Hercules, CA, USA). The following published primer sequences were used for the reactions [20]. GOLPH3, sense (5′-CTCCAGAAACGGTCCAGA AC-3′) and antisense (5′-CCACCAGGTTTTTAGCTAATC G-3′); GAPDH, sense (5′-CTGACTTCAACAGCGACACC-3′) and antisense (5′-TGCTGTAGCCAAATTCGTTG-3′). Denaturation at 95°C for 30 s was followed by 40 annealing cycles of 20 s at 60°C. Expression data was normalized to the geometric mean of a GAPDH housekeeping gene.

### Western blotting

The four matched pairs of PDAC tumor tissues and adjacent non-tumor tissues were harvested and lysed in 50 mM Tris (pH 7.5), 100 mM NaCl, 1 mM EDTA, 0.5% NP40, 0.5% Triton X-100, 2.5 mM sodium orthovanadate, 10 μM protease inhibitor cocktail, and 1 mM phenylmethylsulfonyl fluoride (PMSF). Equal amounts of protein were electrophoretically separated on a 10% SDS-polyacrylamide gel and transferred to polyvinylidene fluoride (PVDF) membranes (Millipore; Bedford, MA, USA). The membranes were incubated at 4°C overnight with anti-human GOLPH3 rabbit monoclonal antibody (1:200; Abgent; San Diego, CA, USA). GOLPH3 expression was detected with horseradish peroxidase (HRP) conjugated goat anti-rabbit IgG secondary antibody (1:2000; Bioss; Shanghai, China) using an electrochemiluminescence (ECL) kit (Keygene; Nanjing, China). Anti-β-actin mouse monoclonal antibody (1:2000; KangCheng; Shanghai, China) was used as a loading control.

### Immunohistochemistry

Immunohistochemical staining was performed on 109 FFPE PDAC tissue samples using an EnVision Kit (DAKO; Hamburg, Denmark) according to the manufacturer’s instructions. Briefly, FFPE sections (4 μm thick) were deparaffinized in xylene, rehydrated in decreasing concentrations of ethanol, and rinsed in phosphate buffered saline (PBS). Antigen retrieval was carried out by microwave treatment in 10 mM citrate buffer (pH 6.0). Endogenous peroxidase activity was quenched in 3% hydrogen peroxide for 10 min. The sections were incubated with primary anti-GOLPH3 rabbit polyclonal antibody (1:200; Abgent) at 4°C overnight, followed by incubation with ready to use EnVision HRP-IgG secondary antibody for 30 min. Staining was developed using 3, 3′-diaminobenzidine (DAB) as a chromogen substrate. The nuclei were counterstained with Mayer’s hematoxylin.

Immunohistochemical staining was evaluated independently by two pathologists (Zhi Li and Ling Xue). The level of GOLPH3 staining was based on the proportion of positively stained tumor cells (area of staining) and the intensity of staining. The following staining scores were applied: Intensity [0 (no staining), 1 (weak staining; light yellow), 2 (moderate staining; yellow brown), 3 (strong staining; brown color)]; the proportion positive tumor cells [0 (no positive tumor cells), 1 (<10% positive tumor cells), 2 (10%–35% positive tumor cells), 3 (35%–70% positive tumor cells), 4 (>70% positive tumor cells)]. The final immunoreactivity score (staining index, SI) was calculated as the product of the staining intensity and staining area scores, giving SI values of 0, 1, 2, 3, 4, 6, 8, 9, or 12, as previously described [21, 22]. The optimal cutoff values were SI ≥ 6 to define tumors with high GOLPH3 expression, and SI ≤4 to define tumors with low GOLPH3 expression.

### Statistical analysis

All statistical analyses were performed using SPSS v. 16.0 statistical software (SPSS Inc.; Chicago, IL, USA). Pearson’s Chi-square (χ^2^) test, Fisher’s exact test and Spearman’s rank correlation were used to analyze the correlations between GOLPH3 expression and clinicopathologic features in patients with PDAC. Patient survival was evaluated using the Kaplan-Meier method and compared using log-rank test. Univariate and multivariate Cox regression analyses were performed to analyze the survival data. A *P*-value <0.05 was considered statistically significant.

## Results

### Clinicopathologic characteristics of patients with PDAC

The 109 patients with PDAC included 68 males and 41 females with a median age of 61 years (range: 28–78 years). The histological grades and clinical stages were classified according the to the tumor-node-metastasis staging system for pancreatic cancer (pTNM), as defined by the American Joint Committee on Cancer (AJCC). This showed that there were 15 cases of stage I, 50 cases of stage II, 22 cases of stage III, and 22 cases of stage IV with liver metastasis. Of these, 7 had well-differentiated tumors, 69 had moderately-differentiated tumors and 33 had poorly-differentiated tumors. The clinicopathologic characteristics of all 109 cases are listed in Table 1.

**Table 1:** **Correlations between GOLPH3 expression and clinicopathologic features in patients with pancreatic ductal adenocarcinoma (PDAC)**

		Expression of GOLPH3		
Clinicopathological feature	Total	Low (n = 30, 27.5%)	High (n = 79, 72.5%)	***P***value (χ ^2^ test)
**Age (years)**				
<61	49	16 (32.7%)	33 (67.3%)	0.278
≥61	60	14 (23.3%)	46 (76.7%)	
**Gender**				
Male	68	18 (26.5%)	50 (73.5%)	0.751
Female	41	12 (29.3%)	29 (70.7%)	
**Tumor location**				
Head	87	25 (28.7%)	62 (71.3%)	0.573
Body/tail	22	5 (22.7%)	17 (77.3%)	
**Clinical stage (pTNM)**				
I	15	7 (46.7%)	8 (53.3%)	0.006*
II	50	6 (12.0%)	44 (88.0%)	
III	22	7 (31.8%)	15 (68.2%)	
IV	22	10 (45.5%)	12 (54.5%)	
**Histological differentiation**				
Well	7	2 (28.6%)	5 (71.4%)	0.949
Moderate/poor	102	28 (27.5%)	74 (72.5%)	
**Size**				
≤2 cm	17	7 (41.2%)	10 (58.8%)	0.170
>2 cm	92	23 (25.0%)	69 (75.0%)	
**T classification**				
T1	5	2 (40.0%)	3 (60.0%)	0.021*
T2	25	10 (40.0%)	15 (60.0%)	
T3	48	6 (12.5%)	42 (87.5%)	
T4	31	12 (38.7%)	19 (61.3%)	
**N classification**				
Absent	56	20 (35.7%)	36 (64.3%)	0.049*
Present	53	10 (18.9%)	43 (81.1%)	
**Liver metastasis**				
Absent	87	20 (22.7%)	67 (77.3%)	0.035*
Present	22	10 (47.6%)	12 (52.4%)	
**Resectability**				
Radical resection	69	19 (27.5%)	50 (72.5%)	0.997
Palliative resection	40	11 (27.5%)	29 (72.5%)	
**Vital status**				
Dead	97	27 (27.8%)	70 (72.2%)	0.836
Alive	12	3 (25.0%)	9 (75.0%)	

*Statistically significant; pTNM: tumor-node-metastasis classification pancreatic cancer staging.

### GOLPH3 is upregulated in pancreatic ductal adenocarcinoma

**Figure 1:**
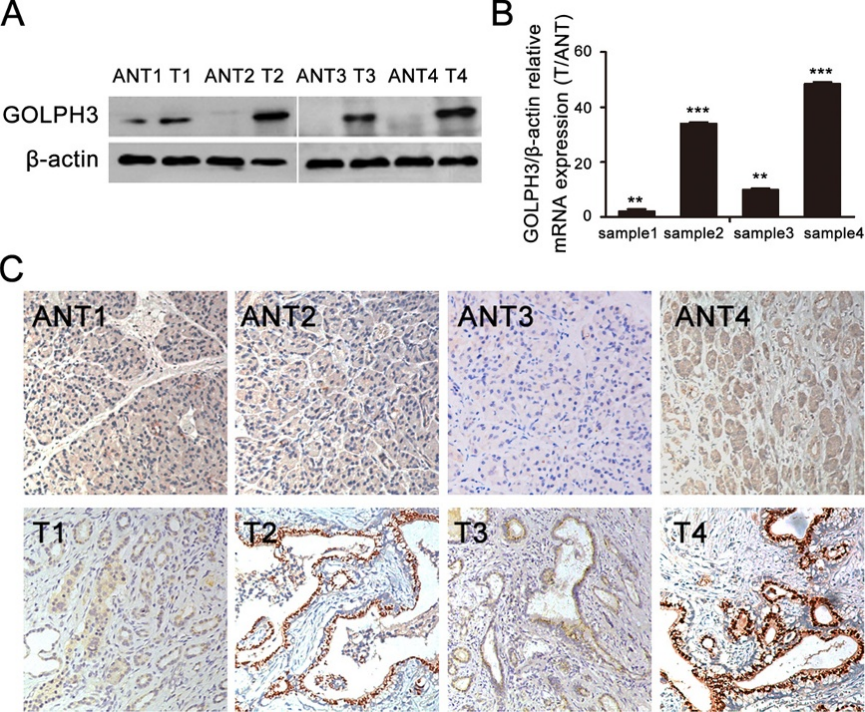
**Expression of GOLPH3 is upregulated in pancreatic ductal adenocarcinoma (PDAC). (A)** Western blots show the expression levels of GOLPH3 protein in four PDAC tumor tissues (T) and their paired adjacent noncancerous tissues (ANT); β-actin was used as loading control. **(B)** Real-time qPCR analysis shows the expression levels of GOLPH3 mRNA in PDAC tumor tissues relative to their paired adjacent noncancerous tissues (T/ANT). Values are given as a ratio of β-actin expression. **P < 0.01; ***P < 0.001. **(C)** Immunohistochemical staining shows the levels and localization of GOLPH3 protein in the four PDAC tumor tissues compared to their paired adjacent no cancerous tissues; (magnification, ×200).

Real-time qPCR and Western blot analysis showed that the expression levels of GOLPH3 mRNA and protein, respectively, were markedly higher in all four PDAC lesions than in their paired adjacent noncancerous tissues (Figure 1A and B).

Immunohistochemistry confirmed that GOLPH3 protein was upregulated in the four PDAC lesions compared to their matched no cancerous tissues (Figure 1C). Furthermore, the immunohistochemical staining patterns revealed that GOLPH3 was mainly localized in the cytoplasm of the tumor cells. None or weak cytoplasmic staining was detectable in the adjacent noncancerous ductal epithelial cells, and no positive staining was found in stoma tissue of tumor and non-tumor tissues.

### GOLPH3 overexpression was correlated with clinical features in PDAC

The immunohistochemical staining results showed that 79/109 (72.5%) of the PDAC cases had a high level of GOLPH3 expression (SI ≥6), whereas 30/109 (27.5%) had a low level of GOLPH3 expression (SI ≤4). These data are given Table 1.

The correlations between GOLPH3 protein expression and clinicopathologic features in PDAC, including age, gender, clinical stage, differentiation status and metastasis were analyzed by Chi-square (χ^2^) test. The results showed that GOLPH3 expression was significantly correlated with clinical stage (*P* = 0.006), T classification (*P* = 0.021), N classification (*P* = 0.049) and liver metastasis (*P* = 0.035); whereas no significant correlations were found with age, gender, tumor location, histological differentiation grade, tumor size and tumor resectability.

### Relationship between GOLPH3 expression and prognosis in PDAC patients

**Figure 2:**
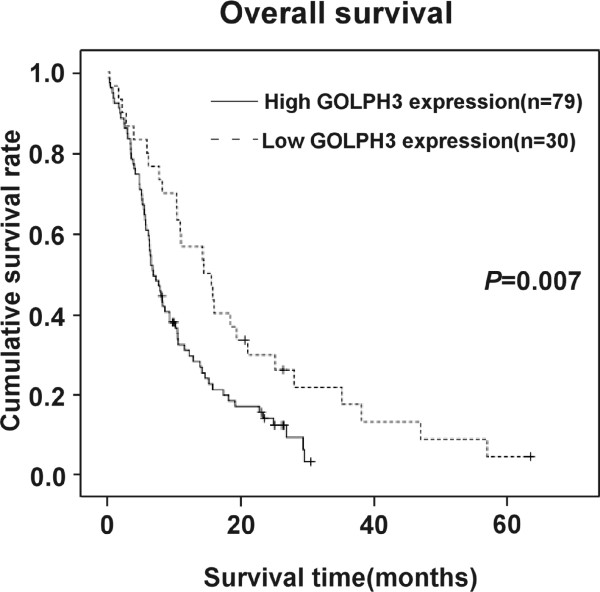
**GOLPH3 expression is correlated with overall survival in patients with PDAC.** The Kaplan-Meier survival curves show the statistical differences in survival times between PDAC patients with high levels of GOLPH3 expression and those with low levels of GOLPH3 expression. *P*-values were calculated by log-rank test.

To determine whether GOLPH3 expression might be a prognostic predictor in PDAC, we examined GOLPH3 expression levels and the clinical follow-up information in all 109 patients of PDAC by Kaplan-Meier analysis and log-rank test. 97 patients died during the follow-up period, whereas 12 patients were still alive at the end of follow-up. The crude and adjusted relative risks of all-cause mortality in these 109 patients were assessed by univariate and multivariate analyses (Table 2; Figure 2). The results showed that the mean survival time in patients with high levels of GOLPH3 expression (9.9 ± 0.9 months, n =79) was significantly shorter than that in patients with a low level of GOLPH3 expression (18.6 ± 2.9 months, n =30) (P = 0.007). Furthermore, the cumulative 3-year survival rate was markedly higher in the low GOLPH3 expression group (17.3% ± 7.4%) compared to that in the high GOLPH3 expression group (3.0% ± 2.8%); These results indicated that patients with high levels of GOLPH3 expression have a worse prognosis than those with low levels of GOLPH3 expression.

**Table 2:** **Univariate and multivariate analyses of prognostic parameters for survival in patients with pancreatic ductal adenocarcinoma (PDAC)**

	Univariate analysis				Multivariate analysis			
Prognostic parameter		95% CI				95% CI		
	RR	Lower	Upper	***P***value	RR	Lower	Upper	***P***value
**Expression of GOLPH3** (low vs. high)	1.915	1.186	3.092	0.008*	2.733	1.578	4.732	0.000*
**Gender** (male vs. female)	0.977	0.643	1.484	0.913	1.008	0.647	1.571	0.972
**Age** (<61 vs. ≥61)	1.295	0.865	1.938	0.209	1.319	0.840	2.073	0.229
**Tumor location** (head vs. body/tail)	0.882	0.521	1.493	0.640	0.619	0.349	1.100	0.102
**Pathologic differentiation** (well vs. moderate/poor)	1.402	0.610	3.223	0.426	0.760	0.308	1.874	0.760
**Size** (≤2 cm vs. >2 cm)	1.940	1.095	3.437	0.023*	1.557	0.841	2.883	0.159
**T classification** (T1 vs. T2 vs. T3 vs. T4)	1.187	0.941	1.496	0.148	1.001	0.658	1.521	0.998
**N classification** (absent vs. present)	1.075	0.718	1.611	0.725	0.608	0.386	0.957	0.032*
**Liver metastasis** (absent vs. present)	1.288	0.779	2.130	0.324	1.002	0.319	3.152	0.997
**Clinical stage** (I vs. II vs. III vs. IV)	1.243	1.014	1.523	0.036*	1.420	0.776	2.598	0.255
**Treatment** (radical vs. palliative)	2.444	1.609	3.710	0.000*	2.828	1.745	4.584	0.000*

Univariate analysis and multivariate analyses were analyzed by Cox regression.

^*^Statistically significant; RR: Relative Risk; 95% CI: 95% confidence interval.

**Figure 3:**
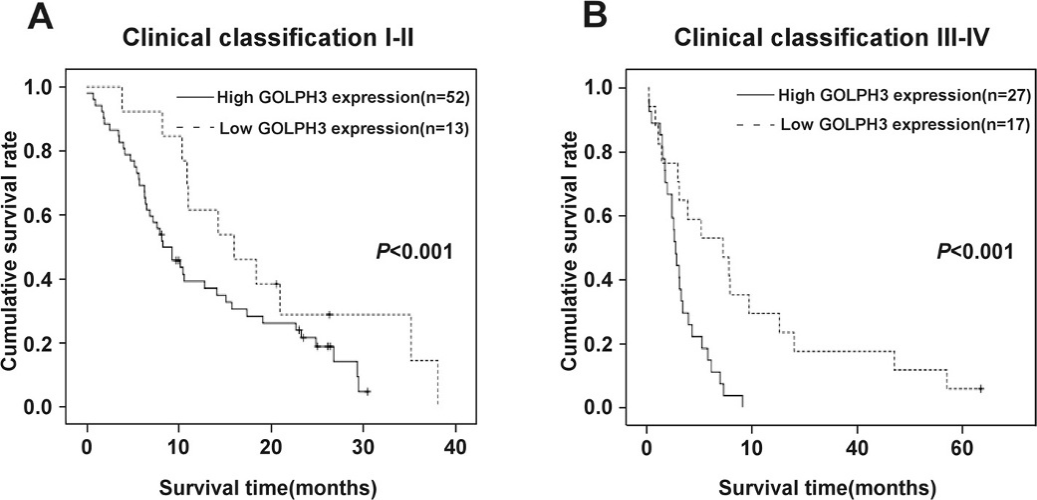
**Overall survival is lower in PDAC patients with high GOLPH3 expression, independent of clinical stage.** Kaplan-Meier survival curves show the statistical differences in overall survival between PDAC patients with high and low expressions of GOLPH3 according to clinical stage: **(A)** early clinical stage (I–II) subgroup; **(B)** advanced clinical stage (II–IV) subgroup. *P*-values were calculated by log-rank test.

**Figure 4:**
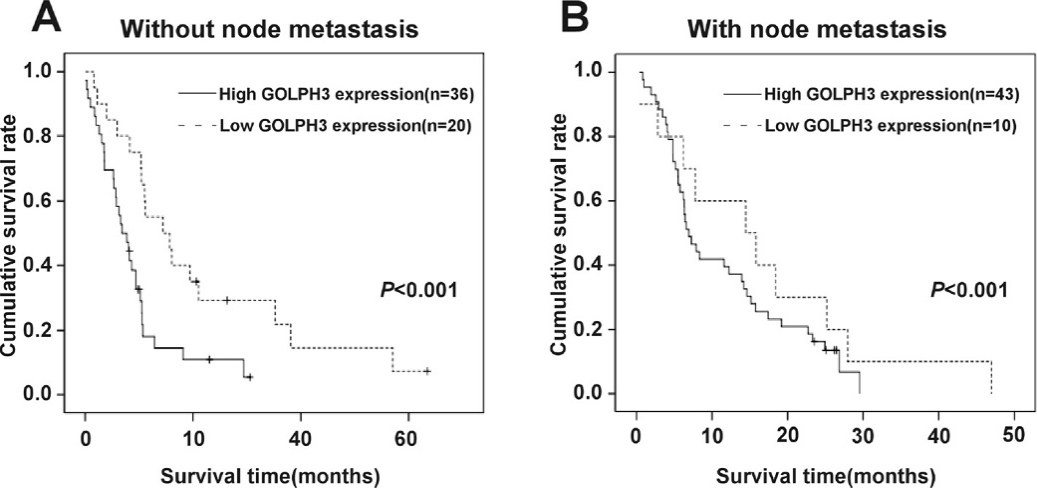
**Overall survival is lower in PDAC patients with high GOLPH3 expression, independent of lymph node metastases status.** Kaplan-Meier survival curves show the statistical differences in overall survival between PDAC patients classified according to lymph node metastasis status and GOLPH3 expression: **(A)** patients without lymph node metastasis; **(B)** patients with lymph node metastasis. *P*-values were calculated by log-rank test.

**Figure 5:**
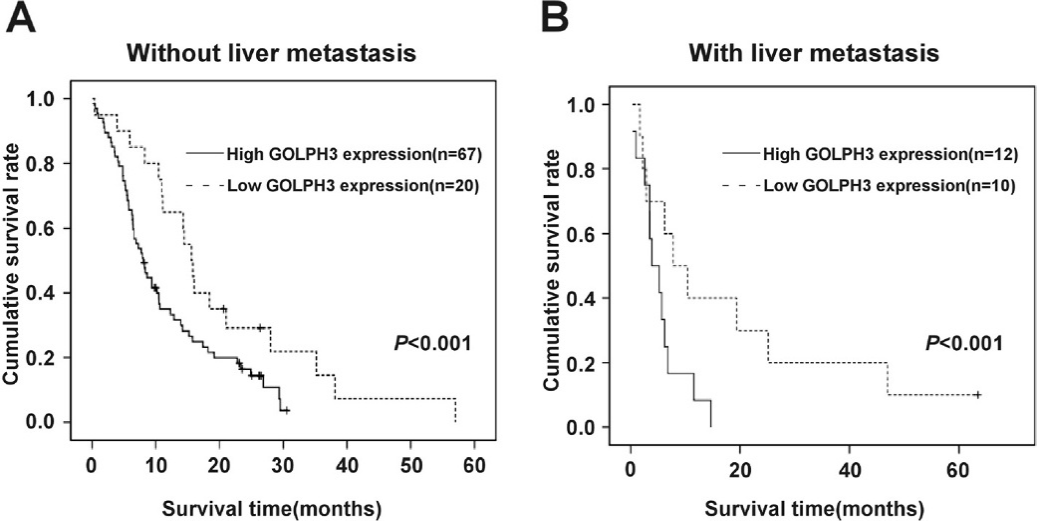
**Overall survival is lower in PDAC in patients with high GOLPH3 expression, independent of liver metastasis status.** Kaplan-Meier survival curves show the statistical differences in overall survival between PDAC patients classified according to liver metastasis status and of GOLPH3 expression: **(A)** patients without liver metastasis; **(B)** patients with liver metastasis. *P*-values were calculated by log-rank test.

We also examined the mean survival times in patient subgroups with different clinical stages or PDAC and with or without lymph node and liver metastasis. The results of Kaplan-Meier analyses showed that patients in the high GOLPH3 expression group with both early (I–II) and advanced clinical stage (III–IV) of PDAC had significantly shorter survival times compared to those in the low GOLPH3 expression group (both *P* < 0.001; Figure 3A and B). Similar results were found between patients in the high and low GOLPH3 expression groups, with and without lymph node metastasis (both *P* < 0.001; Figure 4A and B), with and without liver metastases (*P* < 0.001; Figure 5A and B).

Univariate and multivariate analyses were performed to determine the relative risk of prognostic parameters for the 109 cases of PDAC (Table 2). The Cox proportional hazards model indicated that the expression level of GOLPH3 protein, N classification and tumor resectability were all independent prognostic factors for poor overall survival in patients with PDAC.

## Discussion

Ductal adenocarcinoma accounts for more than 90% of pancreatic malignancies. Early and aggressive local invasion, a high incidence of recurrence, and poor response to chemotherapy and radiotherapy contribute to the poor outcome [3, 23]. The strongest prognostic indicators for long-term patient survival in PDAC are negative margin status, tumor size and absence of lymph node metastases [7, 24].

Recent studies have suggested that GOLPH3 expression may correlate with the growth, proliferation and survival of cancer cells. Overexpression of GOLPH3 was shown to promote cell growth and proliferation in 1205 LU melanoma cells *in vitro*, and enhance tumor growth in xenotransplanted human melanoma and NSCLC cell lines *in vivo* [19]. Depletion of GOLPH3 with small interfering RNA (siRNA) was found to suppress the transformation, proliferation and clonogenic growth in tumor cell lines [18, 19]. GOLPH3 expression has been identified as a prognostic and predictive marker in various human cancers, including breast cancer, oral tongue cancer and esophageal squamous cell cancer [15–17]. However the association between GOLPH3 expression and the clinicopathologic features and patient prognosis in PDAC is unknown.

In this study, we found that GOLPH3 expression was upregulated in PDAC tissues at both the mRNA and protein level compared to adjacent non-tumor tissues. For further confirming the oncogenic role of GOLPH3 in human cancer univariate and multivariate analyses were used and our results revealed that overexpression of GOLPH3 in PDAC was correlated with pTNM stage, lymph node metastases and liver metastases. Furthermore, patients with a high level of GOLPH3 expression had significantly shorter survival times compared to those with a low level of GOLPH3 expression. Stratification survival analysis and Cox regression indicated that high expression of GOLPH3 was an independent factor of poor prognosis, and a potential prognostic indicator in patients with PDAC, suggesting a possible link between the biological function of GOLPH3 and the progression of PDAC. However, χ^2^ tests found no significant difference between GOLPH3 expression and the vital status of patients with PDAC. Tissue samples from more cases of PDAC may be required to determine whether there is a relationship between these two factors. Further mechanistic studies will be proceeding to clarify the role of GOLPH3 in the development of PDAC and to provide insights for new therapeutic targets.

Mammalian target of rapamycin (mTOR) is a serine/threonine protein kinase that exists in two complexes: mTORC1, which is sensitive to rapamycin, and mTORC2. Previous studies have demonstrated that GOLPH3 could regulate tumorigenicity by enhancing activation of mTOR signaling in human cancer cells, thereby influencing cell growth, cell size and proliferation [19]. A potential synergistic link between down regulation of GOLPH3 and rapamycin remains to be elucidated.

Upregulation of GOLPH3 and an increase in proliferation and tumorigenicity has been correlated with the Akt-FoxO1 signaling pathway in breast cancer cells [15]. Overexpression of GOLPH3 confers resistance to killing by DNA-damaging agent in Hela cells. Interference with the GOLPH3/MYO18A pathway significantly impaired cell survival after DNA damage, suggesting that small-molecule inhibitors of the pathway may have therapeutic utility [25]. The exact mechanism following DNA damage through Golgi response to regulate cell survival and function of GOLPH3 in pancreatic cancer as well, will be explored in our further studies.

## Conclusion

This study demonstrated that overexpression of GOLPH3 was associated with poor survival in patients with PDAC, suggesting that GOLPH3 expression may serve as a novel prognostic biomarker and a potential molecular therapeutic target in PDAC.
